# Ex Vivo Biomechanical Assessment of Various Repair Techniques in a Rabbit Calcaneal Tendon Avulsion Model: Application of Polycaprolactone Plate

**DOI:** 10.3390/vetsci10040289

**Published:** 2023-04-12

**Authors:** Zheng Huidan, Jinsu Kang, Namsoo Kim, Suyoung Heo

**Affiliations:** Department of Veterinary Surgery, College of Veterinary Medicine, Jeonbuk National University, 79 Gobong-ro, Iksan 54596, Republic of Korea

**Keywords:** calcaneal tendon rupture, tendon repair, three-loop pulley, tendon plating, polycaprolactone plate, rabbit

## Abstract

**Simple Summary:**

Tendon injuries are a disease that can occur in small animals and may require surgical correction. There are various suture methods for primary tendon repair, and techniques to reinforce these suture methods are currently being researched. This is the first study to apply a plate made of polycaprolactone (biodegradable, biocompatible, and non-toxic implantable biomaterial) to tendon plating. In addition, the polycaprolactone plate was compared biomechanically with conventional tendon reinforcement methods including titanium plates.

**Abstract:**

This study was aimed at evaluating the biomechanical properties and gapping characteristics of tendon repair methods using a combination of a three-loop pulley (3LP) pattern, a titanium plate, and a polycaprolactone (PCL) plate in a rabbit gastrocnemius tendon (GT) model (*n* = 50). GTs were randomly assigned to five groups (*n* = 10/group). Transected GTs were repaired with a 3LP pattern alone or in conjunction with an epitendinous suture (ES), a 5-hole 1 mm PCL plate, a 5-hole 2 mm PCL plate, or a 5-hole 1.5 mm titanium plate. The yield, peak, and failure force, as well as the occurrence and force of 1-mm and 3-mm gapping were examined. The mean yield, peak, and failure force of the 3LP + titanium plate group were higher than that of other groups. The biomechanical properties of a 3LP + a 2 mm PCL plate were similar to 3LP + ES constructs in this model. In all specimens in all groups, 1 mm gap formation was observed. The frequency of 3 mm gap formation was 70% and 90% in the 3LP + 2 mm PCL plate group and the 3LP + 1.5 mm titanium plate group, respectively. Additional studies evaluating PCL plates to determine the effect on the healing and blood supply of tendon are needed.

## 1. Introduction

Tendon injuries account for approximately 0.7% of all musculoskeletal diagnoses in dogs [1]. In particular, tendon injuries arise from chronic overstretching of tendon, laceration, acute trauma or penetrating wounds that require surgical correction [2]. The most common tendon injury requiring surgical correction is partial or complete rupture of the calcaneal tendon (CT) [2]. CT consists of three tendinous structures, each of which is the superficial digital flexor tendon, gastrocnemius tendon (GT), and common tendon of the biceps femoris, gracilis, and semitendinosus muscles [3]. Complete rupture accounted for 26.7% to 42.8% of CT injuries, partial rupture of the superficial digital flexor tendon for 22.2%, and isolated GT rupture for 20% [4,5]. To provide strong repair that resists gap formation at the anastomotic site and to support the tendon during healing are the goal of tendon repair [6]. In veterinary medicine, primary tendon repair has been performed using a variety of core suture methods. The mechanical properties of these methods have been extensively investigated and it has been found that the three-loop pulley (3LP), locking loop, and Krackow techniques are consistently superior to other methods [6,7,8,9,10,11].

The strength of the repair site has been reported to be directly proportional to the number of suture strands across the repair site [12]. When a suture is placed on the epitenon, the strength of the repair is increased by 10% to 50% compared to the core suture alone [13]. The epitendinous suture (ES) pattern minimizes tendon gapping, improves the biomechanical strength of repair, and reduces the cross-sectional area of the repaired tendon [13,14]. Ex vivo canine studies have shown that the ES pattern prevents the occurrence of gap formation before failure, and the 3LP technique significantly increases the ultimate tensile strength of tenorrhaphies by 133% [15].

Additional reinforcement methods for primary repairs, such as synthetic meshes, epithelial sutures, and tendon plating, have been described [15,16,17,18,19,20,21,22,23,24,25,26]. There is some evidence that tendon plating may be a superior method to using the core suture alone to maintain the tendon’s position during healing [16,17,19]. In addition, tendon plating is one of the surgical methods to minimize time in external coaptation and provide an extremely strong form of internal fixation [19]. A material that has biocompatibility and does not compromise the vasculature and matrix of the tendon is required, and for this purpose, there is a previous study in which a tendon plate made of poly-l-lactic acid was applied to the deep digital flexor tendon of a horse in an ex vivo biomechanical experiment [19].

Polycaprolactone (PCL) is biodegradable, biocompatible, non-toxic material that has been widely used as an implantable biomaterial for a variety of applications including drug delivery, tissue engineering and implants [27,28]. In a previous report in human medicine, a customized PCL membrane prototype exhibited good biocompatibility, biodegradability, and mechanical properties [28]. Based on these papers, in this study, we used PCL as the material for the plate and applied it to tendon plating. To the best of our knowledge, the use of PCL plates for tenorrhaphy has not yet been described or biomechanically evaluated.

The objectives of this study were to determine the biomechanical properties and gapping characteristics of various tendon repair methods (3LP alone, 3LP with ES, 3LP with a 1-mm PCL plate, 3LP with a 2-mm PCL plate, and 3LP with a 1.5-mm veterinary cuttable plate). The tensile strength (yield, peak, and failure forces) and gap formation were recorded to determine any differences between the repair methods. We hypothesized that 3LP with a PCL plate (1 mm and 2 mm) would be biomechanically superior to 3LP alone or 3LP with ES, but inferior to 3LP with a 1.5-mm veterinary cuttable plate.

## 2. Materials and Methods

### 2.1. Specimen Preparation

Fifty pelvic limbs obtained from rabbits (3.5 to 4.3 kg) were included in this study. The rabbits were euthanized for reasons unrelated to this study. There was no evidence of related musculoskeletal disorders. All components of the common CT were isolated individually. The gastrocnemius muscle, originating from the supracondylar eminence on the caudodistal femur and inserted into the tuber calcanei, was dissected carefully. The superficial digital flexor tendon was transected 1 cm proximal to its musculoskeletal junction and removed distal to the calcaneal tuberosity using a 10 Bard–Parker blade. Isolated specimens were wrapped in saline (0.9% NaCl)-soaked gauze and stored at −20 °C. This study was approved by the institutional animal care and use committee (IACUC) of Jeonbuk National University (Number: JBNU 2022-063). The specimens were stored at room temperature (21 °C) for 12 h prior to testing. The GTs were transected using a number 10 Bard–Parker blade on a hard and durable calibrated millimeter iron ruler surface at a 1.5-cm distance proximal to the calcaneal tuberosity.

### 2.2. PCL Plate Production

In this study, PCL (704105-100G; Sigma Aldrich, St. Louis, MO, USA) was used in pellet form. The 3-D file of the standard triangle language format of a titanium plate (Able Locking Straight Plate, Able Inc., Jeonju, Republic of Korea) was used to create the PCL plate. The 3-D modeling software (Meshmixer 3.5, Autodesk, San Rafael, CA, USA) was used to model the five-hole plate. The wrap solidify effect (region: outer surface, carve holes: enabled, and minimum hole size: 10 mm) and smoothing (median smoothing method, kernel size: 10 mm) were applied to the 3-D mesh of the plate.

**Figure 1:**
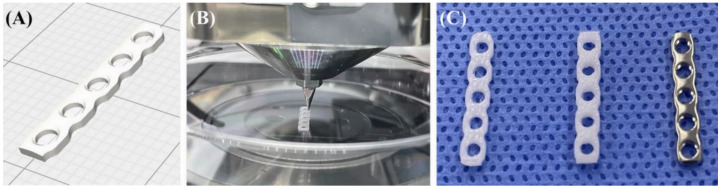
Design and production of polycaprolactone (PCL) plate. (**A**) Design image of the PCL plate. (**B**) PCL plate production process via a dispensing-type 3-D bioprinter. (**C**) From the left, 1-mm PCL plate, 2-mm PCL plate, and 1.5-mm titanium plate, respectively.

A G-code-based script was written by an organ regenerator slicer (ROKIT Healthcare Inc., Seoul, Korea) to print the PCL plate. A dispensing-type 3-D bioprinter (Dr. Invivo 4D6; ROKIT Healthcare Inc., Seoul, Republic of Korea) was used to reconstitute the plate. PCL plates were printed in two groups: 1-mm thickness and 2-mm thickness (Figure 1). The width of the plate was set to 3 mm, the same as the 1.5-mm titanium plate used in the experiment. The printer nozzle size is 0.4 mm and pre-warming is performed for 20 min before the printer proceeds, and the temperature is raised to 80 °C to melt the PCL. The melted PCL was extruded, printing was performed on a culture dish (11090, Duksan General Science, Seoul, Republic of Korea), and the bed temperature under the culture dish was maintained at 33 °C. The wall thickness was 0.8 mm. The slicer settings were as follows: 0.1-mm layer height, 100% infill density, single-line pattern, 7-mm/s printing speed, and 10-mm/s traveling speed.

### 2.3. Tendon Repair Groups

**Figure 2:**
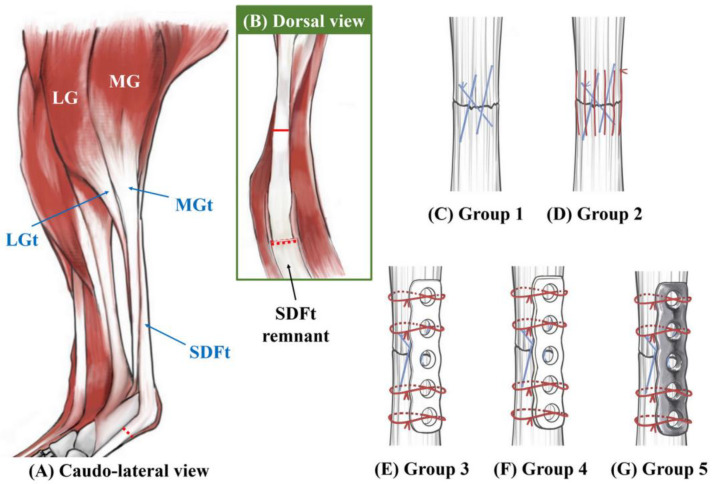
(**A**) Diagram illustrating the rabbit GT in a cadaveric model. (**B**) The superficial digital flexor tendon has been removed. The transected GT was repaired with a 4-0 polydioxanone suture in a three-loop pulley pattern alone (**C**) or in conjunction with an epitendinous suture (**D**), a five-hole 1-mm polycaprolactone (PCL) plate (**E**), a five-hole 2-mm PCL plate (**F**), or a five-hole 1.5-mm titanium plate (**G**). Abbreviations: LG: lateral head of the gastrocnemius, LGt = lateral gastrocnemius tendon, MG = medial head of the gastrocnemius, MGt = medial gastrocnemius tendon, and SDFt = superficial digital flexor tendon.

The specimens were randomly assigned to one of five equally sized experimental groups (*n* = 10 tendons/group) using a randomization software (http://www.random.org/, accessed on 1 July 2022). The tendons resected from the same rabbit were not classified to the same group. All 3LP and ES suture patterns were performed using a 4-0 monofilament polydioxanone (PDS* Plus, Ethicon Inc., Raritan, NJ, USA) with a swaged-on 1/2-circle taper needle. The plates were placed so that they spanned the ends of the two tendons and held in place by suturing the tendon and perimysium through the hole in the plate with a 4-0 monofilament polydioxanone. Referring to the previous literature, the five sutures were tied through the plate using the figure-of-8 pattern [19] (Figure 2).

According to the tendon repair method, the specimens were classified into the following five groups: The 3LP group specimens were repaired using the 3LP technique alone. The 3LP + ES group specimens were repaired using a 3LP technique with the addition of ES sutures. The 3LP + 1-mm PCL plate group specimens were repaired using the 3LP technique with the addition of tendon plating (1-mm thickness, 3-mm width PCL plate). The 3LP + 2-mm PCL plate group specimens were repaired first with a 3LP technique, followed by the addition of tendon plating (2-mm thickness, 3-mm width PCL plate). The 3LP + 1.5-mm titanium plate group specimens were repaired with a 3LP technique and tendon plating using a titanium plate (1.5-mm thickness, 3-mm width; Able Locking Straight Plate; Able Inc., Jeonju, Korea). All tendinous repairs were performed by a single practitioner (Z.H.) under the observation of experienced surgeons (J.-S.K. and S.-Y.H). During the test, a saline solution was administered using a spray to keep all specimens moist.

### 2.4. Biomechanical Evaluation

**Figure 3:**
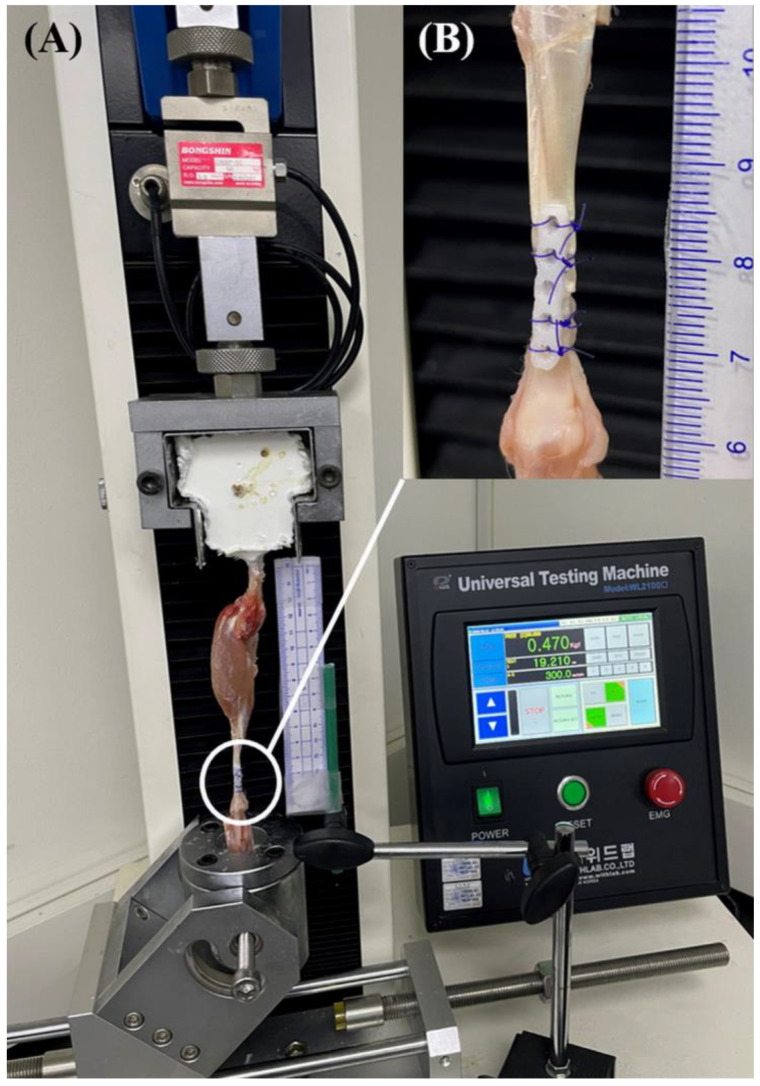
(**A**) Mechanical testing apparatus with a repaired superficial digital flexor tendon specimen within the custom testing jig attached to the material testing machine. (**B**) Magnified photographic image of a transected tendon repaired with a three-loop pulley pattern in conjunction with a five-hole 2-mm PCL plate.

All experiments were performed using a Universal Testing Machine (WL2100C, Withlab Inc., Gunpo, Korea) at room temperature (21 °C). The proximal part of the femur and the proximal part of the musculoskeletal junction were placed in each structure and fixed using an industrial resin. In the process of fixing the specimen using a resin, to mimic the load application of tendon repair with an extended splint in vivo, repaired specimens were positioned with the calcaneus in a vertical orientation. A mobile phone (iPhone 12; Apple Inc., Cupertino, CA, USA) was mounted on a fixed-angle tripod at a 20-cm distance from the repair site. A calibrated ruler was axially aligned with each tendon within the field of the camera to allow for gap formation assessment. Each test was filmed in high definition at 120 frames/s in the slow-motion mode of the mobile phone (Figure 3).

After the initial calibration, a preload of 1 N was applied to ensure a consistent resting length among specimens, and then tension was applied until failure at a distraction rate of 10 mm/min. The testing software (UTM Plus version 1.0016.1201; Withlab Inc., Gunpo, Republic of Korea) collected load (newtons) and displacement (mm) data at a frequency of 100 Hz. The yield, peak, and failure forces were evaluated from the load-placement curve generated in the testing software. The yield force is defined as the point at which there is a detectable non-linear deformation on the load–displacement curve. The peak force is defined as the maximum force measured during each experiment. The failure force was defined as the load at which a suture was broken or pulled through the tendon tissue or a load at which the load displacement curve decreased sharply. The definition of each force is based on a previous study [15]. A single-study practitioner (Z.H.) visually documented the failure method for testing and reviewed the videographic data.

After videographic data analysis, gap formation was assessed using video recordings, using the smallest distance between the tendon ends at the repair site to measure 1-mm and 3-mm gaps. To calculate the load applied when each gap occurred, the time at which the gap occurred was cross-referenced with the load data. If the constructs failed before an identifiable gap was formed, no gapping was reported.

### 2.5. Statistical Analysis

The data were assessed for parametric distribution using the Shapiro–Wilk test for normality. Continuous variables were normally distributed and are described as the mean ± SD. Pairwise comparisons of least squares mean were conducted using Scheffe’s adjustment for multiple comparisons. A *p*-value < 0.05 is considered to be significant. Statistical analyses were conducted using standard software (SPSS version 26.0, IBM Corp., Chicago, IL, USA).

## 3. Results

### 3.1. Load Data

All constructs were successfully repaired and biomechanically tested using all specimens included in the final statistical analysis. Load data were collected and material tests were performed without technical errors.

There was no statistically significant difference in the mean yield force between the 3LP group and the 3LP + 1-mm PCL plate group (*p* = 0.670) and between the 3LP + ES group and the 3LP + 2-mm PCL plate group (*p* = 0.997), the 3LP + 1-mm PCL plate group, and the 3LP + 2-mm PCL plate group (*p* = 0.054). The mean yield force of the 3LP + titanium plate group was higher than that of other groups, with a statistically significant difference. The mean peak force for the 3LP + titanium plate group was higher than that for the other groups. All pairwise comparisons differed (*p* = 0.015), except for the means of the 3LP + ES and 3LP + 2 mm PCL plate groups, which did not (*p* = 0.990). There was no statistically significant difference in the mean failure force between the 3LP group and the 3LP + 1 mm PCL plate group (*p* = 0.191), among the three groups (the 3LP + ES group, the 3LP + 1-mm PCL plate group, and the 3LP + 2-mm PCL plate group) (*p* = 0.060). The mean failure force for the 3LP + 1.5-mm titanium plate group was higher than that for the other groups (Table 1).

### 3.2. Gap Formation Data

A 1-mm gap formation was observed in all specimens in all groups. There was no difference in the force application for a 1-mm gap formation between the 3LP group and the 3LP + 1 mm PCL plate group and between the 3LP + ES group and the 3LP + 2 mm PCL plate group, and among the three groups (the 3LP + ES group, the 3LP + 1 mm PCL plate group, and the 3LP + 2 mm PCL plate group). Force application to cause a 1-mm gap between the 3LP group and the 3LP + ES group, between the 3LP group and the 3LP + 2-mm PCL plate group, between the 3LP group and the 3LP + titanium plate group, and between the 3LP + 1-mm PCL plate group and the 3LP + titanium plate group was different. A 3-mm gap formation was not observed in the 3LP and the 3LP + 1-mm PCL plate groups. There was a difference in force application for a 3-mm gap formation among the 3LP + ES, 3LP + 2-mm PCL plate, and 3LP + 1.5-mm titanium plate groups (Table 2).

## 4. Discussion

This study aims to investigate the biomechanical properties and occurrence of gap formation when using 3LP technology alone or with various reinforcement methods (including PCL plates) in a rabbit tendon laceration model. To the best of author’s knowledge, this is the first study to report a biomechanical assessment of tendon plating using a PCL plate.

The Achilles tendon rupture in dogs is a relatively uncommon injury seen in veterinary clinics [1]. Surgical treatment is most common option with the goal of restoring the anatomical length and providing adequate strength to allow healing [1]. Therefore, various studies on surgical method about tendon repair have been conducted, and many studies have been conducted on not only the suture method but also the fixation method that can be supplemented with suture [2,4,6,7,8,9,10,11,12,15,17,24,25,26].

Rabbits are most often utilized in tendon defects or tenotomy repair studies because of their availability, ease of diagnostic imaging, low housing costs, wide access to dedicated biological products, and reasonably sized grafts [29,30,31]. In addition, there is a previous study that applied the tendon plating method to the calcaneal tendon repair study in rabbits [17]. Therefore, in this study, we compared various tendon repair techniques using a rabbit model.

Among the five groups, as hypothesized previously, the 3LP + titanium plate group was superior in terms of yield, peak, and failure forces. However, contrary to our hypothesis, the 3LP + 1-mm PCL plate group was not superior to the 3LP + ES group in terms of yield, peak, and failure forces. Instead, there was no significant difference in biomechanical properties between the 3LP + 2-mm PCL plate group and the 3LP + ES group in terms of yield, peak, and failure forces.

The critical components for achieving successful tendon repair are the resistance to gap formation and the strength of the primary repair. Gaps greater than 3 mm impede healing and predict repair failure between 21 and 42 days after surgery [32]. Currently, postoperative immobilization for 3 weeks is recommended to reduce the risk of repair failure [4,33,34]. However, treatment options such as external coaptation are associated with a high degree of postoperative morbidity, and immediate active controlled rehabilitation after surgical treatment is recommended in human medicine [35,36].

Through internally protecting sutures positioned at the two ends of the tendon, tendon plating can reduce the requirement for external immobilization [37]. In addition, the multiple areas of fixation provided by tendon plating can reduce the risk of cutting the suture through the tissue, and studies in human medicine revealed that the application of an internal fixation plate increases the tensile strength of tenorrhaphy repairs [38,39,40]. However, the disadvantage of titanium plates is that a second surgery is required for implant removal after the tendon is healed [41]. This removal is caused by concerns about the constant irritation created by the rigid plate attached to the flexible tendon [23]. The devitalization of the tendon due to ischemic injury from multiple suture bites is another potential disadvantage [42].

In this study, we developed a PCL plate to alleviate the limitations of titanium plate applications. PCL exhibited a slow degradation rate. It gradually loses its strength and is absorbed slowly over a period of 2 years [27]. Therefore, there is no need to remove the implant unless fatal complications such as infection occur. Additionally, PCL has mechanical properties that are easy to fine-tune to obtain better soft tissue response compared to titanium [43]. It has received considerable attention in bone tissue engineering applications owing to its mechanical properties. Therefore, they are commonly used for long-term implants [44,45]. This has the potential to provide mechanical safety while minimizing the stimulation applied to tendons, which are flexible tissues. In this study, when 3LP and a 2-mm thick PCL plate were applied, similar biomechanical properties were exhibited despite relatively fewer suture bites compared with 3LP and ES. Furthermore, PCL biocompatibility may be helpful for tendon healing.

PCL can be incorporated with other materials easily to further formulate tissue response. It has been reported that improved osteoblast adhesion, spread to the PCL surface, and increased cell adhesion are occurred in active screen plasma surface modification [46]. Furthermore, the properties of PCL as a drug delivery device can further enhance the effects of platelet-rich plasma (PRP) injection. In studies on horse and rats, it has been reported that PRP increases the tendon strength and improves the organization of the collagen network [47,48,49,50].

This study had some limitations. First, the ex vivo nature of this study cannot accurately represent the inflammatory process and degenerative changes in clinical cases. In addition, it was not possible to evaluate various biological changes or biotoxicity that PCL plate can induce in vivo. Second, we tested constructs to simulate acute clinical failure in which tendon rupture occurred in the immediate perioperative period. For this purpose, we used axial distraction for failure, without cyclic loading. It cannot accurately represent the complex forces applied to the limb during gait in live animals. Third, the number of cases was limited, so we could not conduct an experiment on a control group in which the strength test was performed without resecting the tendon. Fourth, PCL plate requires additional cost and time to design and produce. Due to the time delay required for design and production, PCL plate is not suitable for patients requiring immediate tendon repair. Fifth, since this study was conducted using rabbit cadavers, the same results cannot be guaranteed in other species. Therefore, further studies are required for application in dogs and cats. Sixth, comparative experiments on the shape and width of PCL plates were not conducted. Through further research, it is necessary to study a plate with a biomechanical design considering the shape of tendon, not the same shape as the existing titanium implant. Since the width of the plate can also make a difference in biomechanical results, studies on various thicknesses are required. Finally, no experiments on the optimal thickness of the PCL plate were conducted. For clinical application, biomechanical evaluation at a thickness other than the 1-mm or 2-mm thickness conducted in this study is required. In the case of this study conducted in rabbits, there was no significant difference in the thickness and size of the tendon according to each specimen. However, when applied to other animals, especially in dogs, it is necessary to consider the length as well as the thickness and width depending on the breed and age. In this case, it may be necessary to manufacture customized implants for each patient’s tendon for optimal treatment.

## 5. Conclusions

In conclusion, this study is the first to demonstrate the effectiveness of a PCL plate as a reinforcing material in a 3LP suture pattern compared with other methods for rabbit tendon repair. Our results revealed that the biomechanical properties of a 3LP + 2-mm PCL plate were similar to those of the 3LP + ES constructs in this model. Therefore, a PCL plate can be considered an alternative to the ES pattern in conjunction with a 3LP suture pattern. Additional studies evaluating PCL plate augmentation in vivo are needed to determine its effect on tendinous healing and blood supply in tendons prior to clinical use.

## Figures and Tables

**Table 1:** Mean ± SD yield, peak, and failure forces for canine gastrocnemius tendons that underwent transverse tenotomy and repair with various suture patterns.

Suture Pattern	Mean Yield Force, N	Mean Peak Force, N	Mean Failure Force, N
3LP	14.97 ± 2.28 ^a^	18.03 ± 4.35 ^a^	16.88 ± 3.76 ^a^
3LP + ES	31.44 ± 11.36 ^b^	41.01 ± 10.17 ^b^	38.33 ± 9.81 ^b^
3LP + 1-mm PCL plate	19.92 ± 3.58 ^a,c^	28.75 ± 5.23 ^c^	26.47 ± 5.06 ^a,b^
3LP + 2-mm PCL plate	30.13 ± 5.44 ^b,c^	39.29 ± 4.00 ^b^	37.30 ± 5.27 ^b^
3LP + 1.5-mm titanium plate	52.20 ± 9.01 ^d^	67.55 ± 9.18 ^d^	60.36 ± 14.00 ^c^

Values with different superscript letters are significantly (^a–d^ *p* < 0.05) different.

**Table 2:** Force and occurrence of 1-mm and 3-mm gapping for experimental groups.

Suture Pattern	1-mm Gapping		3-mm Gapping	
	Force, Mean ± SD, N	Frequency (%)	Force, Mean ± SD, N	Frequency (%)
3LP	16.02 ± 3.81 ^a^	10/10 (100%)	N/A	0
3LP + ES	30.09 ± 4.98 ^b,c^	10/10 (100%)	32.97 ± 3.42 ^a^	2/10(20%)
3LP + 1-mm PCL plate	24.71 ± 6.38 ^a,b^	10/10 (100%)	N/A	0
3LP + 2-mm PCL plate	27.27 ± 4.45 ^b^	10/10 (100%)	39.02 ± 2.13 ^b^	7/10(70%)
3LP + 1.5-mm titanium plate	39.35 ± 13.15 ^c^	10/10 (100%)	49.46 ± 6.76 ^c^	9/10(90%)

Values with different superscript letters are significantly (^a–c^ *p* < 0.05) different. N/A; not assessed.

## Data Availability

All the data generated or analyzed in this study are included in this published article.
